# Artificial Intelligence and Natural Photosensitizer-Based Nanopharmaceuticals in Photodynamic Therapy: Advanced Modeling, Data-Driven Optimization, and Translational Perspectives

**DOI:** 10.3390/pharmaceutics18080921

**Published:** 2026-07-27

**Authors:** Renato Sonchini Gonçalves, Emmanoel Vilaça Costa

**Affiliations:** 1 Department of Engineering and Exact Sciences, Setor Palotina, Federal University of Paraná (UFPR), Palotina 85950-000, PR, Brazil; 2 Postgraduate Program in Chemistry, Federal University of Amazonas (UFAM), Manaus 69080-900, AM, Brazil; emmanoelvc@gmail.com

**Keywords:** photodynamic therapy, artificial intelligence, machine learning, natural photosensitizers, AI-driven nanopharmaceuticals, nanoformulation, drug delivery, photosensitizer discovery, QSAR, QSPR, radiomics, translational development, precision medicine

## Abstract

Photodynamic therapy (PDT) is a minimally invasive therapeutic modality based on the interaction between a photosensitizer (PS), light, and molecular oxygen to generate reactive oxygen species (ROS) capable of inducing localized cytotoxicity. Natural products provide a chemically diverse source of photosensitizers, including curcumin, hypericin, hypocrellin, chlorin derivatives, alkaloids, flavonoids, anthraquinones, and other photoactive scaffolds. However, their translational development remains limited by poor solubility, aggregation, instability, variable purity, limited tissue penetration, suboptimal pharmacokinetics, and insufficient formulation readiness. In parallel, artificial intelligence (AI), including machine learning (ML), deep learning (DL), quantitative structure–activity relationship (QSAR) and quantitative structure–property relationship (QSPR) modeling, radiomics, and predictive analytics, is increasingly being applied to photosensitizer discovery, molecular property prediction, nanoformulation optimization, treatment planning, and precision PDT. This critical review evaluates the intersection between AI, natural photosensitizers, nanopharmaceutical development, and PDT, with emphasis on methodological strengths, current limitations, and translational priorities. A PRISMA 2020-inspired search strategy identified 27 studies for qualitative synthesis, comprising 11 review articles and 16 original investigations, while additional seminal references were used for historical and mechanistic contextualization. The analysis indicates that current AI applications in PDT are concentrated around molecular property prediction, QSAR/QSPR modeling, phototoxicity assessment, radiomics, image-guided therapy, and treatment-response prediction, whereas AI-guided exploration of natural photosensitizer chemical space and AI-assisted nanoformulation design remain comparatively underdeveloped. Key barriers include heterogeneous datasets, limited natural-product representation in predictive models, insufficient external validation, weak integration between formulation variables and photodynamic outcomes, and limited consideration of manufacturing and regulatory requirements. This review proposes an integrated AI-enabled translational framework connecting natural-product chemical diversity, photochemical prediction, nanocarrier optimization, precision PDT validation, and clinical implementation.

## 1. Introduction

Photodynamic therapy (PDT) is a minimally invasive therapeutic modality that relies on the interaction between a photosensitizer (PS), the light of an appropriate wavelength, and molecular oxygen to generate reactive oxygen species (ROS), particularly singlet oxygen, capable of inducing localized cytotoxicity. Since its clinical introduction, PDT has emerged as an attractive therapeutic strategy for the treatment of malignant diseases, including cutaneous tumors, pancreatic cancer, peritoneal metastases, melanoma, prostate cancer models, and oral premalignant or malignant lesions, as well as several non-malignant conditions such as dermatological diseases, antimicrobial and periodontal infections, corneal neovascularization, and retinal or fundus diseases. These applications illustrate the broad clinical and translational relevance of PDT, from oncology and dermatology to ophthalmology and oral health, due to its spatial selectivity, reduced systemic toxicity, and potential for repeated applications. The therapeutic efficacy of PDT depends on a complex interplay among photosensitizer properties, light delivery parameters, oxygen availability, and biological responses within the target tissue [1,2,3,4,5,6,7,8,9].

Despite significant advances over the past decades, several limitations continue to restrict the broader clinical implementation of PDT. Many photosensitizers exhibit poor aqueous solubility, limited tumor selectivity, prolonged skin photosensitivity, aggregation-induced quenching, and suboptimal pharmacokinetic profiles. Furthermore, treatment outcomes may be influenced by tumor heterogeneity, oxygen depletion, inadequate dosimetry, and variability in light distribution, highlighting the need for more efficient photosensitizers and improved treatment-planning strategies [2,6,10,11].

In response to these limitations, increasing attention has been directed toward the development of alternative photosensitizers derived from natural sources. Natural products represent a vast reservoir of structurally diverse bioactive molecules that have evolved to perform a wide range of ecological functions, including light absorption and photochemical activity. Several naturally occurring compounds, such as curcumin, hypericin, hypocrellin, chlorophyll-derived pigments, and other photoactive metabolites, have demonstrated promising photodynamic properties, including ROS generation, low dark toxicity, and favorable biocompatibility profiles. Moreover, their intrinsic biological activities may provide additional therapeutic benefits beyond photodynamic action, including anti-inflammatory, antimicrobial, antioxidant, and anticancer effects. Consequently, natural photosensitizers have emerged as attractive candidates for the development of safer and more effective PDT platforms [6,10,12,13,14,15].

Despite their considerable potential, the development of natural photosensitizers for clinically relevant PDT remains challenging. Many natural compounds exhibit poor water solubility, limited chemical stability, low bioavailability, rapid degradation, and insufficient tumor selectivity. In addition, the enormous chemical diversity of natural products creates substantial challenges for the identification, characterization, optimization, and prioritization of candidate photosensitizers. Traditional experimental screening approaches are often labor-intensive, time-consuming, and costly, limiting the exploration of the vast natural chemical space that may contain novel photoactive molecules. These limitations highlight the need for innovative strategies capable of accelerating photosensitizer discovery while simultaneously improving pharmaceutical development and translational feasibility [6,10,12,15,16,17].

Concurrently, artificial intelligence (AI) has emerged as a transformative technology across multiple areas of biomedical research, pharmaceutical sciences, and precision medicine. Machine learning (ML), deep learning (DL), quantitative structure–activity relationship (QSAR) and quantitative structure–property relationship (QSPR) modeling, virtual screening, molecular property prediction, and generative AI approaches are increasingly being employed to accelerate drug discovery and optimize therapeutic development pipelines. By identifying complex patterns within large and multidimensional datasets, these computational tools enable the prediction of biological activity, toxicity, pharmacokinetic behavior, and physicochemical properties, significantly reducing the time and cost associated with conventional experimental workflows. In recent years, AI-driven methodologies have demonstrated remarkable success in molecular design, lead optimization, biomarker discovery, medical imaging, and clinical decision support, highlighting their potential to reshape the future of translational medicine [18,19,20,21].

Within the field of photodynamic therapy, AI applications are rapidly expanding beyond traditional drug discovery frameworks. Computational models have been developed to predict the singlet oxygen quantum yields, photophysical properties, phototoxicity profiles, and structure–activity relationships of photosensitizers, facilitating the identification of promising candidates before experimental validation. In parallel, machine learning and deep learning algorithms have been applied to treatment planning, dosimetry optimization, image-guided PDT, radiomics-based patient stratification, and therapeutic outcome prediction. These advances suggest that AI may contribute not only to the discovery of novel photosensitizers but also to the development of precision PDT strategies capable of improving treatment efficacy, personalization, and clinical decision-making [22,23,24,25,26,27,28,29].

Although substantial progress has been achieved independently in natural photosensitizer research, photodynamic therapy, nanopharmaceutical development, and artificial intelligence, the integration of these domains remains limited, fragmented, and unevenly developed. Existing reviews have generally emphasized either the biological potential of natural photosensitizers, the formulation advantages of nanocarriers, or the broad application of AI in drug discovery and medical imaging. However, fewer studies have critically examined how computational modeling, data-driven optimization, pharmaceutical development, and translational constraints intersect within the specific context of natural product-based PDT. This gap is particularly relevant for Pharmaceutics, because the clinical value of natural photosensitizers depends not only on photochemical activity but also on solubility, stability, manufacturability, delivery efficiency, biological performance, regulatory feasibility, and integration into reproducible therapeutic workflows [12,15,16,17,23,30,31,32].

**Figure 1:**
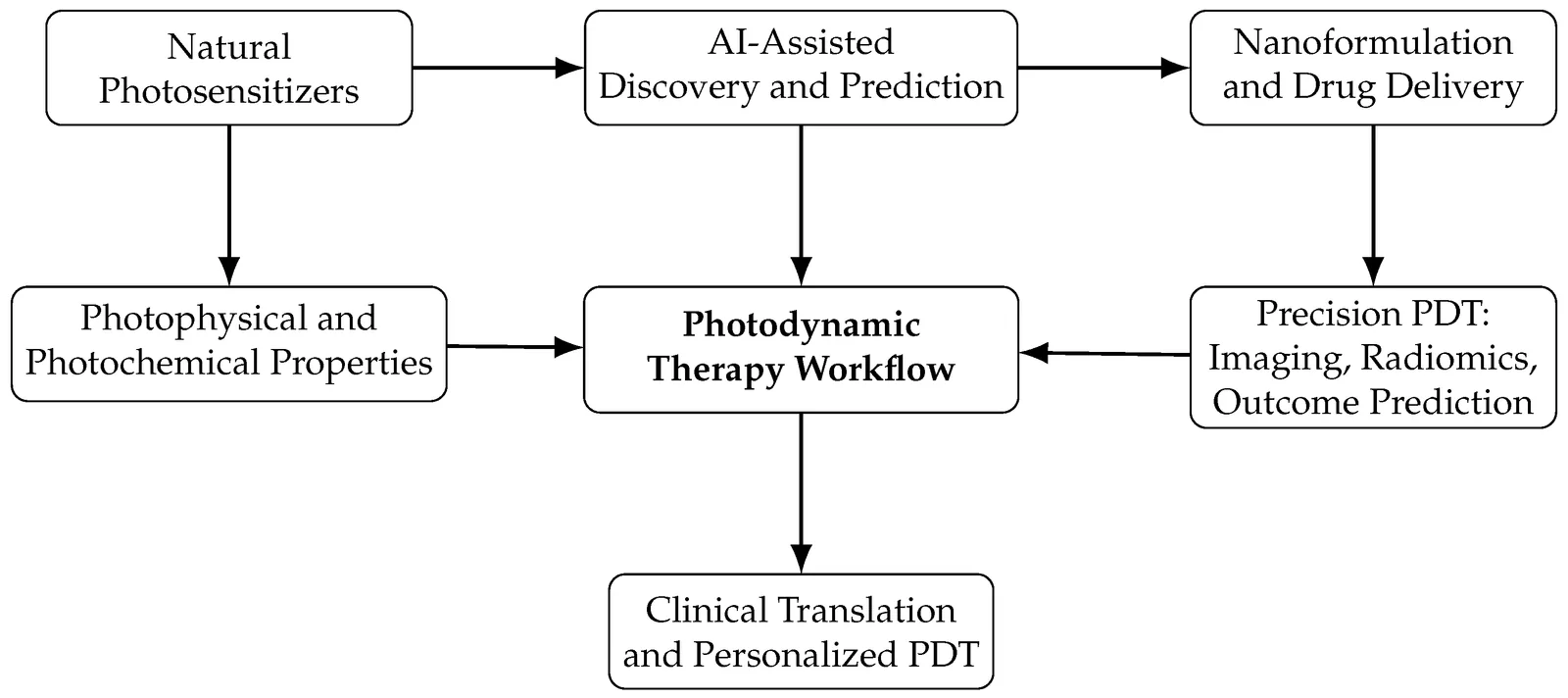
Conceptual framework linking natural photosensitizers, artificial intelligence-assisted discovery and prediction, nanoformulation and drug delivery, precision photodynamic therapy, and clinical translation. The figure emphasizes that natural photosensitizer development should not be viewed as a linear sequence from compound identification to therapy, but as an integrated translational workflow in which molecular diversity, predictive modeling, formulation performance, photophysical behavior, imaging, treatment-response prediction, and clinical feasibility are interdependent. This framework supports the central argument of the review that AI-driven approaches may accelerate PDT development only when connected to pharmaceutical optimization, standardized validation, and clinically relevant implementation strategies.

Therefore, this review was designed not only to summarize the available literature but also to critically evaluate the methodological quality, practical impact, and translational relevance of current approaches at the interface of AI, natural photosensitizers, nanotechnology-enabled delivery systems, and PDT. Particular attention is given to the strengths and limitations of AI-assisted photosensitizer discovery, QSAR/QSPR modeling, phototoxicity prediction, pharmaceutical and nanocarrier optimization, radiomics, image-guided treatment, outcome prediction, and clinical translation. By comparing current methodologies and identifying unresolved challenges, this review aims to define research priorities for the development of AI-driven nanopharmaceutical platforms based on natural photosensitizers. The proposed conceptual framework, illustrated in Figure 1, highlights the need to connect molecular diversity, predictive modeling, formulation science, precision PDT, and translational validation within a unified development pipeline [12,14,15,23,30,31,32,33].

## 2. Literature Search Strategy and Study Selection

This critical review was conducted using a structured literature search strategy inspired by the Preferred Reporting Items for Systematic Reviews and Meta-Analyses (PRISMA 2020 ) framework [34]. Although the objective was not to perform a quantitative systematic review or meta-analysis, PRISMA principles were adopted to enhance transparency, reproducibility, and methodological rigor during study identification, screening, and selection.

The literature search was performed in the PubMed and Scopus databases. Search terms were designed to capture studies at the intersection of PDT, natural photosensitizers, AI, pharmaceutical development, and nanotechnology-enabled delivery systems. Combinations of keywords related to “photodynamic therapy”, “photosensitizer”, “natural products”, “curcumin”, “hypericin”, “hypocrellin”, “chlorin”, “nanoparticles”, “nanocarriers”, “drug delivery”, “nanoformulation”, “pharmaceutical development”, “artificial intelligence”, “machine learning”, “deep learning”, “QSAR”, “QSPR”, “radiomics”, “dosimetry”, “treatment planning”, and “precision medicine” were employed using Boolean operators adapted to each database, following the transparency principles recommended for structured review reporting [34].

Because the objective of this review was to provide a critical and translational analysis rather than a purely descriptive mapping of the literature, the search results were re-examined with particular attention to four priority domains: (i) AI-assisted photosensitizer discovery and molecular property prediction; (ii) AI-supported pharmaceutical development and nanoformulation optimization; (iii) imaging, radiomics, dosimetry, and treatment-response prediction in PDT; and (iv) emerging or underexplored natural photosensitizers with potential relevance for nanopharmaceutical development. This additional level of screening was used to ensure that the final discussion addressed not only the presence of studies within the field but also their methodological robustness, practical limitations, translational relevance, and contribution to future AI-driven PDT workflows [23,30,31,32,33,35,36,37,38].

Only studies published between 2015 and 2026 were considered eligible for screening. A total of 1088 records were identified through database searching, including 594 records from PubMed and 494 records from Scopus. After the removal of 265 duplicate records, 823 unique records remained. Application of the predefined publication-year filter resulted in the exclusion of 81 records, yielding 742 studies for title and abstract screening, in accordance with the structured reporting logic adopted for this PRISMA-inspired review process [34].

Following the screening process, 85 articles were selected for full-text assessment. Studies were excluded when they were not directly related to PDT, did not involve AI-based methodologies or natural photosensitizers, or lacked sufficient methodological relevance to the objectives of this review. After eligibility assessment, 27 studies met all inclusion criteria and were included in the qualitative synthesis. These studies comprised 11 review articles and 16 original investigations, following the structured eligibility and reporting principles adopted from the PRISMA 2020 framework [34].

In addition to the primary corpus obtained from database searches, seminal and complementary references were identified through citation tracking and manual snowballing. These sources were used to strengthen the historical, mechanistic, pharmaceutical, and computational context of the review, particularly when recent advances in AI-assisted photosensitizer modeling, singlet oxygen prediction, radiomics, treatment-response prediction, dosimetry, and nanopharmaceutical development were relevant to the critical interpretation of the selected studies. However, these complementary references were not included in the PRISMA quantitative counts, because they were used for contextual discussion rather than as part of the predefined PubMed/Scopus corpus [1,2,3,4,5,6,10,34].

During revision, the literature coverage was re-examined to ensure that the discussion included the most relevant and recent studies related to modern AI approaches, pharmaceutical development, nanoformulation strategies, precision PDT, and emerging natural photosensitizer systems. Particular attention was given to whether the selected and complementary studies allowed for critical comparison of methodological strengths, limitations, validation strategies, translational maturity, and future research priorities. This approach was adopted to avoid a purely descriptive synthesis and to align the review more closely with the scope of AI-driven nanopharmaceutical development [24,25,30,31,33,35,36,37,39].

**Figure 2:**
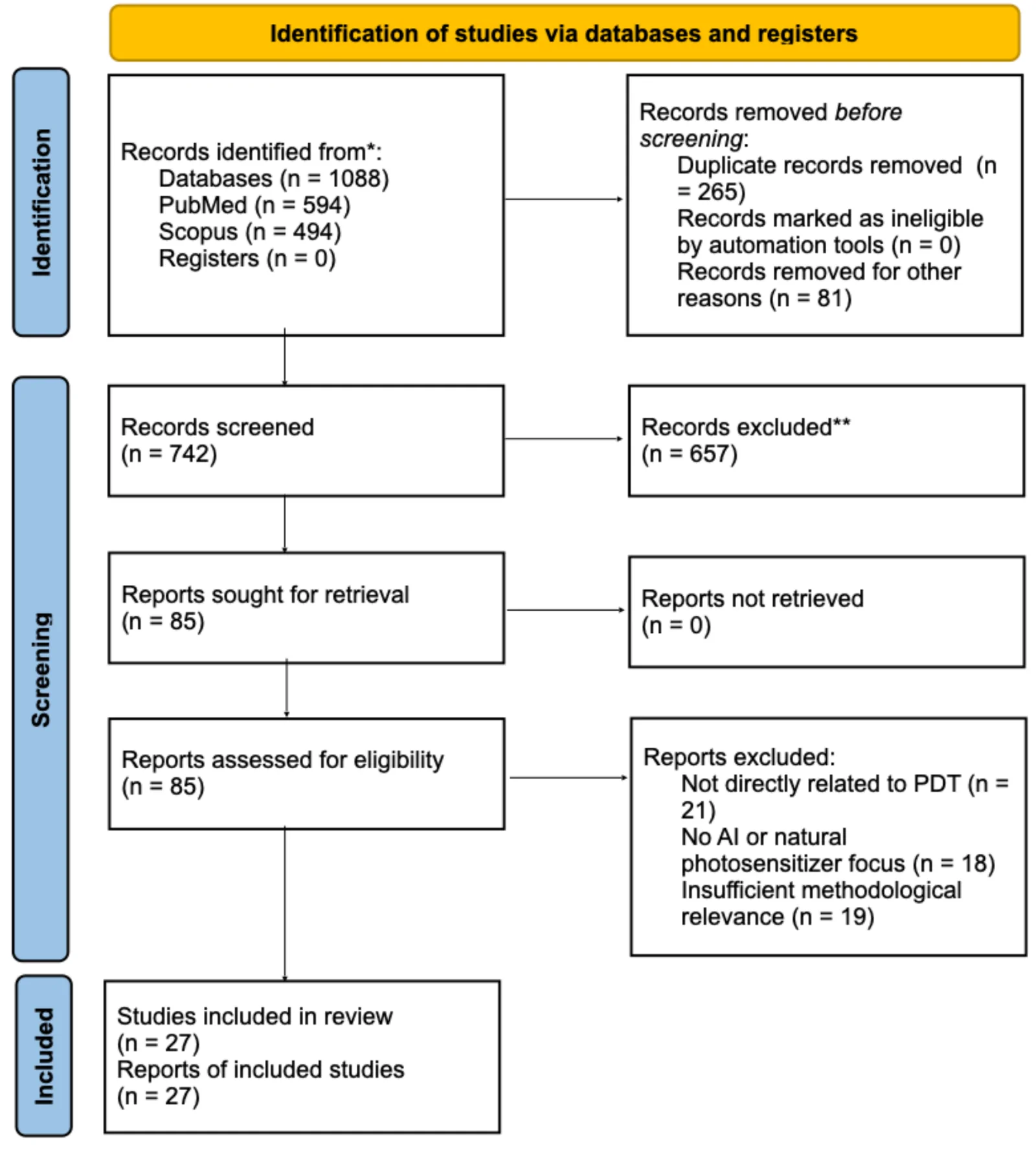
PRISMA 2020-inspired flow diagram summarizing the literature search and study selection process. From 1088 records identified in PubMed and Scopus, 27 studies met the eligibility criteria and were included in the qualitative synthesis. The 657 records excluded after title/abstract screening were removed because they were not directly related to photodynamic therapy, did not present an artificial intelligence or natural photosensitizer focus, lacked relevant photosensitizer, nanoformulation, or translational PDT content, or were outside the conceptual scope of the review. The reasons for exclusion at the eligibility stage are detailed separately in the corresponding box. * Records identified are reported separately for each database. ** All records were excluded manually during title/abstract screening, and no automation tools were used.

The complete study selection process is summarized in the PRISMA flow diagram presented in Figure 2, following the reporting structure recommended by the PRISMA 2020 statement [34].

## 3. Overview of Included Studies

The final corpus comprised 27 studies that met the predefined eligibility criteria, including 11 review articles and 16 original investigations. Rather than representing a mature and consolidated research field, the corpus reveals an emerging and heterogeneous literature in which natural photosensitizer research, AI-based modeling, nanotechnology-enabled delivery, and precision PDT have developed at different speeds. The review articles generally provide broad conceptual syntheses of natural photosensitizers, nanotechnology, or AI in PDT, whereas the original investigations are more narrowly focused on specific computational models, delivery platforms, imaging workflows, or biological applications. This imbalance indicates that the field is still transitioning from parallel descriptive domains toward integrated, data-driven, and translationally oriented nanopharmaceutical development [12,13,14,15,16,17,30,31,40,41,42].

The included studies were organized into four major thematic areas: (i) natural photosensitizers and their photodynamic applications; (ii) AI-assisted photosensitizer discovery and molecular property prediction; (iii) AI-supported pharmaceutical development and nanotechnology-based delivery; and (iv) AI-enabled precision PDT, including radiomics, image-guided therapy, dosimetry support, and treatment-response prediction. However, these categories should not be interpreted as equally developed. AI-assisted molecular prediction and imaging-based outcome modeling are supported by more explicit computational workflows, whereas AI-guided pharmaceutical development and nanoformulation optimization remain comparatively underdeveloped and are often discussed as future opportunities rather than demonstrated experimental pipelines [23,30,31,32,33,35,36,37,38].

A critical comparison of the original studies shows important methodological differences in data source, model purpose, validation level, and translational maturity. QSAR/QSPR and ML-based studies provide useful proof-of-concept strategies for predicting photodynamic activity, singlet oxygen generation, drug photosensitization, and phototoxicity, but they frequently rely on limited chemical datasets, heterogeneous experimental endpoints, and incomplete external validation. Imaging-based and radiomics studies are closer to clinical decision support, yet they remain constrained by small cohorts, single-center designs, retrospective data structures, and variability in imaging protocols or treatment parameters. These limitations reduce model generalizability and highlight the need for standardized reporting, external validation, explainable modeling, and prospective evaluation before AI tools can be reliably incorporated into PDT workflows [23,32,33,35,36,37,38].

From the perspective of natural photosensitizers, the corpus remains strongly concentrated around curcumin, hypericin, and hypocrellin-based systems. These compounds are scientifically relevant because they combine photodynamic potential with intrinsic biological activities, but their repeated predominance also reveals a narrow exploration of natural-product chemical space. Other photoactive classes, including chlorophyll derivatives, pheophorbides, alkaloids, anthraquinones, flavonoids, furanocoumarins, and marine-derived metabolites, remain poorly represented in AI-assisted PDT studies. This limited chemical diversity is a major weakness for predictive modeling, because AI systems trained on restricted or structurally biased datasets may fail to capture the broader photophysical and pharmaceutical behavior of natural photosensitizer candidates [15,16,17,43,44,45,46,47,48,49,50].

The pharmaceutical and nanotechnological evidence also presents an uneven level of maturity. Several studies demonstrate that nanogels, glyconanoparticles, targeted nanoparticles, polydopamine-coated nanorods, and protein-based assemblies can improve the solubility, stability, targeting, uptake, and photodynamic performance of natural photosensitizers. Nevertheless, most formulation studies remain experimentally driven and do not yet incorporate systematic AI-supported optimization, quality-by-design logic, predictive release modeling, manufacturability assessment, or a regulatory-oriented comparability analysis. Therefore, the principal translational gap is not simply the lack of nanocarriers, but the absence of integrated datasets connecting formulation variables, physicochemical properties, irradiation conditions, biological endpoints, and therapeutic outcomes [16,17,30,48,49,50,51,52].

Overall, the available literature suggests that AI has the potential to influence multiple stages of the PDT development pipeline, but the current evidence is stronger for computational feasibility than for validated translational implementation. The main opportunity for the field is to move beyond isolated demonstrations of molecular prediction, formulation development, or imaging analysis toward integrated AI-driven nanopharmaceutical workflows. Such workflows should combine natural-product chemical diversity, curated photophysical datasets, formulation and nanocarrier descriptors, standardized irradiation parameters, biological validation, imaging biomarkers, and clinically meaningful outcomes. The characteristics of the studies included in the qualitative synthesis are summarized in Table 1, while the following sections provide a more critical analysis of their methodological strengths, limitations, and translational implications [23,30,31,32,33,35,36,37,38].

The comparative profile presented in Table 1 reveals three important patterns. First, the natural photosensitizer literature remains predominantly focused on a limited number of compounds and delivery platforms, particularly curcumin-, hypericin-, and hypocrellin-based systems, whereas broader natural-product chemical diversity remains insufficiently explored. Second, AI-based studies are concentrated mainly on molecular prediction, phototoxicity assessment, radiomics, image-guided procedures, and outcome prediction, with comparatively fewer examples directly addressing AI-assisted nanoformulation optimization. Third, the translational maturity of the included studies is uneven: several investigations provide proof-of-concept computational or preclinical evidence, but few integrate molecular prediction, pharmaceutical development, standardized irradiation parameters, biological validation, and clinically relevant endpoints within a unified workflow. These patterns support the need for more comparative, data-driven, and pharmaceutically oriented research strategies in AI-assisted natural photosensitizer-based PDT [16,17,23,30,31,32,33,35,36,38].

### Methodological and Translational Appraisal of the Included Studies

A methodological appraisal of the included studies indicates that the current literature remains heterogeneous in both design and translational maturity. Reviews generally provide broad conceptual coverage of natural photosensitizers, AI applications, or nanotechnology-enabled PDT, but they frequently do not compare computational validation strategies, formulation readiness, or clinical implementation barriers in depth. Original computational studies provide more explicit AI workflows, including ML, DL, QSAR/QSPR, radiomics, and phototoxicity prediction; however, many remain limited by small datasets, restricted chemical domains, retrospective data structures, or insufficient external validation. These limitations are particularly relevant because AI models intended for PDT must generalize across different photosensitizer classes, irradiation protocols, biological models, formulation systems, and clinical scenarios [23,30,31,32,33,36,37,38].

From a pharmaceutical perspective, the most important gap is the limited integration between AI methods and formulation development. Several natural photosensitizer studies demonstrate the value of nanogels, nanoparticles, glyconanoparticles, protein assemblies, and multifunctional nanocarriers, but most of these systems are optimized experimentally rather than through predictive or data-driven formulation models. Critical pharmaceutical variables, including particle size, surface charge, loading capacity, release kinetics, aggregation state, stability, rheology, bioadhesion, tissue retention, manufacturability, and batch reproducibility, are rarely incorporated into AI-ready datasets. This weak connection between computational prediction and pharmaceutical development limits the ability to translate promising natural photosensitizers into reproducible nanopharmaceutical PDT platforms [16,17,48,49,50,51,52].

Overall, the evidence base supports the feasibility of AI-assisted PDT but does not yet demonstrate a fully integrated translational pipeline. The most advanced applications are currently found in molecular property prediction, phototoxicity assessment, radiomics, image-guided procedures, and outcome prediction, whereas AI-assisted nanoformulation optimization and regulatory-oriented pharmaceutical development remain comparatively immature. This imbalance justifies the need for future studies that combine curated datasets, standardized photochemical assays, formulation descriptors, biological validation, imaging biomarkers, dosimetry parameters, and clinically meaningful outcomes within unified AI-driven development frameworks [23,30,31,32,33,35,36,38].

## 4. Natural Photosensitizers in Photodynamic Therapy

Natural products have emerged as an important source of photosensitizers for PDT because they offer broad structural diversity, biological multifunctionality, and access to chemical scaffolds that are often absent from conventional synthetic libraries. However, their value for PDT should not be assessed only by their ability to generate ROS under irradiation. From a pharmaceutical and translational perspective, natural photosensitizers must also be evaluated according to aqueous solubility, chemical and photochemical stability, aggregation behavior, purity, reproducibility of the source material, compatibility with scalable formulation processes, delivery efficiency, and safety under dark and irradiated conditions. This broader evaluation is essential because several promising natural compounds show strong photodynamic activity in vitro but fail to progress toward translational use due to unfavorable biopharmaceutical or formulation-related properties [12,13,14,15,16,40,41].

The selected literature indicates that natural photosensitizers encompass multiple chemical classes, including polyphenols, perylenequinones, chlorophyll derivatives, alkaloids, flavonoids, anthraquinones, and other photoactive secondary metabolites. These classes differ markedly in absorption profile, ROS-generation mechanism, lipophilicity, aggregation tendency, subcellular localization, and susceptibility to degradation. Consequently, direct comparison among natural photosensitizers is difficult when studies use different light sources, irradiation doses, solvents, biological models, formulation systems, and endpoints. This methodological heterogeneity limits the identification of structure–performance relationships and reduces the ability to define which natural scaffolds are most suitable for AI-assisted optimization and nanopharmaceutical development [12,13,14,15].

A critical limitation of the current field is that the most frequently investigated natural photosensitizers are also those for which major pharmaceutical barriers remain unresolved. Curcumin, hypericin, and hypocrellin-based systems dominate the selected corpus, but each presents a distinct translational bottleneck. Curcumin is attractive due to its safety profile and biological activity, yet its poor aqueous solubility, rapid degradation, limited tissue penetration, and blue-light dependence restrict broader applicability. Hypericin exhibits stronger photophysical performance and fluorescence properties, but its hydrophobicity, aggregation, and delivery challenges require sophisticated carrier systems. Hypocrellin offers favorable photochemical characteristics and potential for phototheranostics, but the related literature remains comparatively limited and strongly dependent on advanced formulation strategies [16,17,46,47,48,49,50].

Nanotechnology-enabled delivery systems have therefore become central to the development of natural photosensitizers, not merely as auxiliary carriers but as determinants of therapeutic performance. In the selected studies, nanogels, targeted nanoparticles, glyconanoparticles, polydopamine-coated nanorods, and protein-based assemblies were used to improve solubility, stability, cellular uptake, targeting, controlled release, imaging capability, and photodynamic efficiency. Nevertheless, most of these systems remain experimentally optimized by trial and error, with limited use of AI-supported formulation design, predictive modeling, or quality-by-design strategies. This represents a major gap for the scope of AI-driven nanopharmaceuticals, since formulation variables such as particle size, surface charge, encapsulation efficiency, release profile, carrier composition, and biological interactions could be incorporated into predictive models to guide rational development [16,30,48,49,50,51,52].

Another important limitation is the narrow representation of natural-product chemical space in current AI-PDT studies. While curcumin, hypericin, and hypocrellin are valuable model compounds, the limited inclusion of other photoactive natural scaffolds restricts the diversity and predictive power of computational datasets. Emerging classes such as pheophorbides, chlorophyll derivatives, anthraquinones, flavonoids, furanocoumarins, and marine-derived pigments may offer useful photophysical or pharmaceutical properties, but they remain insufficiently integrated into AI-assisted screening and nanopharmaceutical workflows. Expanding the chemical diversity of training datasets will be essential for identifying new natural photosensitizer candidates and avoiding model bias toward a small number of well-studied scaffolds [12,14,15,31].

To support a more comparative and translational interpretation of the natural photosensitizers discussed in this review, Table 2 summarizes the main advantages, pharmaceutical limitations, and AI/nanopharmaceutical priorities associated with the major natural photosensitizer classes. This comparison emphasizes that the development of natural photosensitizers for PDT depends not only on intrinsic photochemical activity but also on formulation readiness, delivery performance, standardization, and compatibility with AI-assisted optimization workflows [12,13,14,15,16,17,30,31].

### 4.1. Curcumin-Based Photosensitizers

Curcumin, a polyphenolic compound primarily isolated from the rhizomes of *Curcuma longa* L., remains one of the most frequently investigated natural photosensitizers in PDT research. Its appeal is supported by a favorable safety profile, low dark toxicity, broad biological activity, and reported anticancer, anti-inflammatory, antimicrobial, and antioxidant effects. However, from a critical translational perspective, curcumin should be considered a pharmacologically attractive but pharmaceutically challenging photosensitizer. Its PDT performance is strongly conditioned by formulation, irradiation wavelength, local concentration, oxygen availability, and biological context, which partly explains the variability observed across experimental studies [12,14,15,16].

The major limitation of curcumin is not the absence of photodynamic activity, but the difficulty of converting this activity into a robust and reproducible therapeutic platform. Curcumin exhibits poor aqueous solubility, low systemic bioavailability, chemical instability under physiological conditions, susceptibility to degradation, and limited tissue penetration because its strongest absorption occurs in the blue region. These properties restrict deep-tissue PDT applications and make curcumin more suitable, at least in its native form, for topical, superficial, antimicrobial, oral, dermatological, or daylight-mediated PDT strategies. Therefore, studies using curcumin should be interpreted according to the intended clinical setting rather than as broadly generalizable evidence for all PDT applications [10,12,15,16].

In addition to curcumin itself, naturally occurring curcuminoids should be considered in the development of curcumin-based photosensitizing systems. The major natural curcuminoids include curcumin, demethoxycurcumin, and bisdemethoxycurcumin (BDMC), which differ in the substitution pattern of the aromatic rings and consequently in their physicochemical, photophysical, and stability profiles. BDMC is particularly relevant because the absence of methoxy groups has been associated with altered polarity, higher relative hydrophilicity, and improved chemical stability compared with curcumin, including lower susceptibility to hydrolytic degradation. From a PDT perspective, these properties may be advantageous because solubility, aggregation state, degradation rate, and photostability strongly influence light absorption, ROS generation, cellular uptake, and reproducibility of photodynamic outcomes. Therefore, BDMC and related natural curcuminoids should not be viewed only as minor constituents of turmeric extracts, but as promising natural derivatives for comparative evaluation, AI-assisted property prediction, and nanoformulation optimization in curcumin-based PDT platforms [43,44,45].

Molecular modification represents one strategy to improve curcumin-based photosensitization. Chlorin e6-curcumin conjugates illustrate how hybrid systems may combine the biological advantages of curcumin with improved photophysical properties derived from chlorin scaffolds. This approach may enhance ROS generation, cellular uptake, and phototoxicity compared with curcumin alone. Nevertheless, such conjugates also raise additional development questions related to synthetic reproducibility, purity, stability, structure–activity relationships, pharmacokinetics, and regulatory classification. Thus, molecular hybridization should be evaluated not only by improved in vitro phototoxicity but also by its ability to generate chemically well-defined, scalable, and formulation-compatible photosensitizer candidates [46].

Nanoformulation has been the most practical route for improving curcumin delivery. Nanoparticles, nanogels, liposomes, polymeric carriers, and multifunctional nanocomposites can increase apparent solubility, protect curcumin against degradation, improve local retention, and support controlled release. In the selected corpus, curcumin-containing nanogel systems illustrate the potential of topical nanopharmaceutical platforms for daylight PDT, particularly when curcumin is combined with other natural bioactive components. However, these systems also demonstrate the need for deeper pharmaceutical analysis, including release kinetics, rheological behavior, stability during storage, penetration or retention in target tissues, reproducibility of preparation, and scalability of the manufacturing process [10,16,48].

From an AI-driven nanopharmaceutical perspective, curcumin is a useful model compound because its limitations are multidimensional and therefore suitable for data-driven optimization. ML, QSAR, and QSPR approaches could be used to predict the photophysical properties, degradation susceptibility, ROS-generation potential, molecular stability, and structure–activity relationships of curcumin derivatives before synthesis. In parallel, AI-assisted formulation models could incorporate carrier composition, particle size, zeta potential, encapsulation efficiency, release profile, viscosity, bioadhesion, and photodynamic response as interdependent variables. At present, however, direct AI-guided optimization of curcumin-based PDT systems remains limited, and future studies should move from empirical formulation screening toward integrated computational–experimental workflows with standardized endpoints and external validation [23,24,25,30,31].

### 4.2. Hypericin-Based Photosensitizers

Hypericin, a naturally occurring perylenequinone predominantly isolated from species of the genus *Hypericum*, is one of the most photophysically attractive natural photosensitizers for PDT. Its high singlet oxygen quantum yield, broad visible absorption, fluorescence properties, and potent light-induced cytotoxicity make it relevant for oncological, antimicrobial, antifungal, and phototheranostic applications. In contrast to curcumin, whose PDT use is often restricted by blue-light dependence and rapid degradation, hypericin offers stronger photochemical performance and better intrinsic suitability for imaging-guided strategies. However, these advantages do not automatically translate into clinical readiness, because hypericin presents major pharmaceutical limitations that require advanced formulation and delivery solutions [12,15,17,55].

The main translational barrier for hypericin is its poor aqueous solubility and strong tendency to aggregate in biological media. Aggregation can reduce effective light absorption, alter fluorescence behavior, impair ROS generation, and compromise reproducibility across experimental systems. In addition, limited bioavailability, nonspecific distribution, and challenges in achieving selective accumulation at target tissues restrict the therapeutic window of free hypericin. Therefore, the development of hypericin-based PDT should be evaluated not only according to phototoxic potency, but also according to formulation stability, aggregation control, delivery selectivity, release behavior, imaging performance, and safety under clinically relevant irradiation conditions [15,17,49,51,52].

The selected studies demonstrate that nanotechnology-based delivery systems can partially address these limitations. Hypericin-loaded glyconanoparticles, targeted nanoparticles, and polydopamine-coated cerium oxide nanorods have been used to improve dispersion, cellular uptake, tumor targeting, and photodynamic efficacy. These systems are particularly relevant to the scope of AI-driven nanopharmaceuticals because their performance depends on multiple interacting variables, including carrier composition, surface chemistry, particle size, charge, drug loading, hypericin aggregation state, release kinetics, and biological microenvironment. Nevertheless, most hypericin nanoplatforms remain optimized empirically, and few studies provide systematic modeling of how formulation parameters determine photophysical behavior and therapeutic response [17,47,49,51,52].

Hypericin is also highly relevant for phototheranostic development because its fluorescence can support visualization of cellular uptake, biodistribution, and treatment localization. This property creates opportunities for integration with AI-assisted image analysis, radiomics, and quantitative fluorescence-based monitoring. However, current evidence remains limited by the lack of standardized image acquisition protocols, quantitative thresholds, longitudinal biodistribution datasets, and externally validated predictive models. As a result, the potential of hypericin for image-guided precision PDT is conceptually strong but still requires more rigorous translational validation before it can support reproducible clinical decision-making [17,31,38,47,49].

From an AI perspective, hypericin-based systems are particularly suitable for data-driven optimization because their limitations involve both molecular and formulation-dependent factors. ML models could be used to predict aggregation propensity, photostability, fluorescence intensity, ROS-generation efficiency, carrier compatibility, and biological response of hypericin derivatives or nanoformulations. In addition, imaging datasets obtained from hypericin fluorescence could be integrated with radiomics and outcome prediction models to support patient-specific PDT planning. The critical requirement for this progress is the creation of curated datasets that connect molecular structure, formulation descriptors, photophysical measurements, irradiation conditions, imaging features, and biological endpoints. Without this integration, hypericin will remain a highly promising but incompletely translated natural photosensitizer [23,24,25,31,38].

### 4.3. Hypocrellin and Related Systems

Hypocrellins are naturally occurring perylenequinone pigments isolated primarily from fungi of the genus *Shiraia*. Hypocrellin A and hypocrellin B have attracted attention as natural photosensitizers because of their visible-light absorption, efficient ROS generation, relatively high singlet oxygen production, and photodynamic activity against cancer cells and microbial pathogens. Compared with curcumin, hypocrellins generally present more favorable photophysical properties; compared with hypericin, they remain less extensively explored and less translationally mature. This intermediate position makes hypocrellins scientifically promising but also highlights the need for more systematic pharmaceutical, computational, and biological evaluations [12,14,15,50].

The principal limitation of hypocrellins is the mismatch between favorable photochemistry and incomplete pharmaceutical readiness. Similar to other hydrophobic natural photosensitizers, hypocrellins may suffer from poor aqueous solubility, aggregation, rapid clearance, limited tumor accumulation, and variable biodistribution. These limitations can reduce effective light absorption, alter ROS generation, and compromise the reproducibility of therapeutic outcomes. Therefore, hypocrellin-based PDT systems should be assessed not only by phototoxicity under optimized experimental conditions but also by stability, carrier compatibility, release behavior, tissue distribution, irradiation depth, and performance under biologically relevant oxygen conditions [10,15,50].

The HSA-based hypocrellin B nanoplatform identified in the selected corpus illustrates both the promise and the complexity of this class. By incorporating hypocrellin into a biocompatible protein-based assembly, the system improved tumor targeting, fluorescence imaging, and NIR-responsive phototheranostic performance. This strategy is valuable because it addresses key barriers associated with hydrophobicity and delivery while enabling integrated imaging and therapy. However, it also raises questions that are central to nanopharmaceutical translation, including batch-to-batch reproducibility, protein–photosensitizer binding stability, loading efficiency, release kinetics, scalability, sterilization compatibility, and predictive control of in vivo performance [50].

Hypocrellin-based phototheranostic platforms are also relevant to precision PDT because they connect molecular photochemistry, nanocarrier design, imaging, and treatment response within a single therapeutic workflow. This creates a natural interface with AI tools, particularly for quantitative image analysis, treatment planning, optical-property estimation, and response prediction. Nevertheless, the current evidence remains largely preclinical and platform-specific. Future studies should generate standardized datasets linking hypocrellin structure, carrier properties, optical absorption, fluorescence behavior, ROS production, irradiation parameters, oxygen status, biodistribution, and therapeutic outcomes. Such datasets would allow AI models to evaluate which formulation and treatment variables most strongly determine efficacy [26,27,28,29,31].

Although hypocrellin-related studies remain less numerous than those involving curcumin or hypericin, this limited literature may represent an opportunity rather than only a weakness. Because hypocrellins combine favorable photophysical properties with substantial formulation challenges, they are well suited for AI-assisted optimization strategies that integrate molecular modeling, ML-based property prediction, and nanocarrier design. However, meaningful progress will require moving beyond isolated demonstrations of phototheranostic efficacy toward comparative studies that benchmark hypocrellin systems against other natural photosensitizers, evaluate formulation robustness, and validate predictive models experimentally. In this sense, hypocrellins may serve as an important test case for the AI-driven development of next-generation natural photosensitizer nanopharmaceuticals [23,24,25,30].

### 4.4. Emerging Natural Photosensitizers and Underexplored Chemical Space

Although curcumin, hypericin, and hypocrellin dominate the current literature on natural photosensitizers, this predominance should be interpreted critically. These compounds have generated valuable experimental evidence, but their repeated use has also narrowed the chemical diversity explored in PDT and may have biased the field toward a limited number of structurally familiar scaffolds. Numerous naturally occurring photoactive compounds, including chlorophyll derivatives, pheophorbides, alkaloids, anthraquinones, flavonoids, furanocoumarins, and marine-derived pigments, have potential relevance for PDT because they may combine visible-light absorption, ROS generation, intrinsic biological activity, and structural diversity. However, their investigation remains fragmented and substantially less developed than that of curcumin- and hypericin-based systems [12,13,14,15].

The limited representation of alternative natural photosensitizers in contemporary AI-PDT studies is a major scientific and computational limitation. AI models are only as informative as the datasets used to train and validate them. If available datasets are dominated by a small number of compound families or by synthetic photosensitizers, predictive algorithms may fail to identify relevant structure–property relationships for natural-product-derived scaffolds. This is particularly problematic for natural compounds, whose photodynamic performance depends not only on chromophore structure but also on stereochemistry, functional-group distribution, lipophilicity, aggregation behavior, degradation profile, and interaction with biological or nanocarrier environments [12,14,15,31].

The underutilization of many natural photosensitizer classes also reflects practical barriers in traditional discovery workflows. Experimental screening of large natural-product libraries is costly, time-consuming, and technically demanding, especially when photophysical characterization, ROS quantification, dark toxicity, phototoxicity, formulation compatibility, and biological validation must be performed under standardized irradiation conditions. In addition, many natural compounds are available only in small quantities, may vary according to biological source or extraction method, and may require purification or structural confirmation before reliable testing. These challenges slow the transition from biodiversity-derived chemical diversity to pharmaceutically meaningful PDT candidates [6,10,12,15].

AI-driven workflows could address part of this bottleneck by prioritizing natural-product candidates before extensive experimental evaluation. ML, QSAR, QSPR, DL, molecular fingerprints, graph-based representations, and generative approaches may be used to predict absorption properties, triplet-state behavior, singlet oxygen generation, photostability, aggregation tendency, phototoxicity risk, and formulation compatibility. However, the practical value of these methods depends on the availability of curated datasets that include negative as well as positive examples, standardized irradiation conditions, experimentally measured photophysical endpoints, and formulation-relevant descriptors. Without such datasets, AI may accelerate screening but not necessarily improve translational decision-making [23,24,25,32,37,39].

Future studies should therefore move from isolated evaluation of individual natural compounds toward systematic AI-assisted exploration of natural photosensitizer chemical space. Integrating biodiversity repositories, natural-product databases, molecular docking or quantum chemical descriptors, photophysical measurements, nanoformulation parameters, and biological endpoints could enable more rational prioritization of new candidates. From a nanopharmaceutical perspective, this strategy should not end at molecular selection; it should also predict whether a candidate is compatible with scalable delivery systems, controlled release, tissue targeting, and clinically realistic light exposure. Such integrated workflows may expand the repertoire of natural photosensitizers and provide a more robust foundation for next-generation AI-driven PDT platforms [18,24,25,30,31,39].

## 5. Artificial Intelligence in Photodynamic Therapy

The integration of AI into PDT represents one of the most significant recent developments in the field, but its current impact should be evaluated critically. Traditional photosensitizer discovery and optimization have relied on synthesis, extraction, photophysical characterization, biological screening, and iterative empirical refinement. Although these approaches remain indispensable, they are slow, costly, and poorly suited to exploring large chemical spaces or multidimensional formulation variables. AI methods, including ML, DL, QSAR/QSPR modeling, molecular descriptor analysis, graph-based learning, radiomics, and predictive analytics, offer an opportunity to prioritize candidate molecules, estimate photochemical behavior, anticipate toxicity, optimize delivery systems, and personalize treatment protocols. However, most AI applications in PDT remain proof-of-concept studies, and their translational value depends strongly on dataset quality, external validation, interpretability, and compatibility with experimental and clinical workflows [18,21,23,32,33,35,36,38].

A critical distinction should be made between AI models that improve scientific understanding and AI models that can support translational decision-making. In photosensitizer discovery, descriptor-based ML and QSAR/QSPR approaches may identify molecular features associated with ROS generation, singlet oxygen quantum yield, absorption profile, phototoxicity, and photosafety. In pharmaceutical development, AI can potentially connect formulation composition, particle size, zeta potential, encapsulation efficiency, release kinetics, stability, and biological response. In clinical PDT, radiomics and DL can integrate imaging and clinical data to support lesion detection, treatment planning, dosimetry estimation, patient stratification, and outcome prediction. Nevertheless, these applications differ substantially in data requirements, validation needs, and regulatory implications, and they should not be treated as equivalent levels of evidence [23,30,31,32,33,35,36,37,38].

The main AI applications identified across the PDT workflow are summarized in Table 3, including photosensitizer discovery, photochemical property prediction, phototoxicity assessment, nanoformulation optimization, image-guided PDT, radiomics, outcome prediction, and clinical decision support. Compared with purely descriptive summaries, the table emphasizes the type of computational approach, the stage of the PDT pipeline, the potential contribution, and the main translational limitations associated with each application. This analytical organization is important because the maturity of AI in PDT is uneven: molecular property prediction and phototoxicity screening are closer to computational implementation, whereas AI-guided nanoformulation optimization and clinically validated decision support remain less developed [23,30,31,32,33,35,36,38].

Taken together, the available evidence indicates that AI can support PDT development at multiple interconnected levels, from molecular discovery and photochemical property prediction to formulation design, imaging analysis, dosimetry support, and clinical decision-making. For the purposes of this review, AI-related applications in PDT are organized into three main areas: (i) photosensitizer discovery and molecular property prediction; (ii) pharmaceutical and nanotechnological optimization; and (iii) precision PDT through radiomics, imaging analysis, treatment planning, and outcome prediction. The following subsections critically discuss these areas with emphasis on methodological strengths, limitations, practical impact, and future priorities for the AI-assisted development of natural photosensitizer-based nanopharmaceuticals [23,31,32,33,35,36,38].

### 5.1. AI-Assisted Photosensitizer Discovery and Molecular Property Prediction

AI-assisted photosensitizer discovery represents one of the most direct and mature applications of data-driven modeling in PDT research, but its current evidence should be interpreted as enabling rather than definitive. Conventional photosensitizer development depends on sequential extraction or synthesis, structural confirmation, photophysical characterization, biological testing, and iterative optimization. This workflow is experimentally robust but inefficient when large libraries of synthetic, semi-synthetic, or natural-product-derived compounds must be evaluated. ML- and descriptor-based models can reduce this burden by prioritizing molecules with favorable absorption profiles, triplet-state behavior, ROS-generating potential, singlet oxygen quantum yield, photostability, low dark toxicity, and suitable predicted biopharmaceutical properties before experimental validation [23,32,33,37].

The computational approaches used in photosensitizer discovery vary substantially in complexity and interpretability. Traditional QSAR/QSPR models rely on molecular descriptors, physicochemical parameters, and regression or classification algorithms to relate chemical structure to photodynamic activity. Molecular fingerprints and similarity-based models can support the rapid screening of structurally related candidates, whereas graph-based learning and deep neural networks may capture more complex structural relationships when sufficient training data are available. More advanced strategies, including generative models and hybrid DFT–ML workflows, could support de novo photosensitizer design and prediction of excited-state or photophysical properties. However, the practical use of these methods in natural photosensitizer discovery remains limited by the small datasets, inconsistent endpoints, and the scarcity of standardized photochemical measurements [22,23,24,25,39].

Among the studies included in this review, Buglak et al. [23] demonstrated the relevance of QSAR/QSPR modeling for predicting photodynamic activity and singlet oxygen generation in BODIPY photosensitizers. This approach is important because singlet oxygen production is a central determinant of Type II PDT efficacy, while its experimental quantification can be laborious, method-dependent, and difficult to scale across large molecular libraries. A key strength of QSAR/QSPR modeling is its interpretability, since molecular descriptors can provide insight into how structural features influence photochemical performance. Nevertheless, models developed for specific synthetic families such as BODIPYs cannot be directly generalized to natural photosensitizers without careful validation, because natural scaffolds may differ substantially in conformational flexibility, aggregation behavior, substituent patterns, and interaction with biological or nanocarrier environments [12,15,22,23].

AI-based phototoxicity and photosafety prediction represent another important component of early-stage screening. Halinkovic et al. [32] developed MLtox as an online platform for phototoxicity prediction, while Hofmann et al. [37] applied AI-based strategies to predict photosensitizing effects of drugs and chemical compounds. These models are valuable because they can identify potential light-induced toxicity before costly biological validation and may reduce late-stage failures. However, photosafety prediction should be distinguished from PDT efficacy prediction. A compound predicted to be phototoxic may not necessarily be therapeutically useful, because clinical PDT requires appropriate light absorption, selective accumulation, controllable ROS generation, acceptable dark toxicity, formulation compatibility, and a feasible route of administration. Therefore, phototoxicity models should be integrated with pharmaceutical and biological filters rather than used as isolated decision tools [31,32,37].

AI-assisted molecular design has also begun to move from risk prediction toward the active optimization of high-performance photosensitizers. Diao et al. [33] reported an ML-assisted strategy for the design of cyanine photosensitizers, illustrating how computational models can guide the selection of molecular features associated with improved photodynamic and theranostic performance. Although cyanine systems are not natural photosensitizers, this study provides an important methodological reference because similar workflows could be adapted to natural-product-derived scaffolds such as curcumin, hypericin, hypocrellin, chlorin derivatives, pheophorbides, anthraquinones, and other underexplored photoactive metabolites. For natural compounds, however, predictive models should also account for extractability, structural variability, chemical stability, derivatization feasibility, and compatibility with nanocarrier systems [12,14,15,33].

Complementary studies reinforce the value of hybrid computational workflows. He et al. [24] developed experimentally validated ML models for predicting singlet oxygen quantum yield, while Gao et al. [25] combined DFT calculations with ML to predict the properties of transition-metal-complex photosensitizers. These examples show that AI can be strengthened by physically meaningful descriptors and experimentally validated endpoints. For natural photosensitizers, similar approaches could combine quantum chemical parameters, molecular descriptors, absorption features, predicted excited-state behavior, solubility estimates, aggregation descriptors, and formulation-relevant variables. This integration would be more useful than purely statistical screening because it would connect molecular design with pharmaceutical feasibility and translational performance [24,25,39].

Despite these advances, several methodological weaknesses limit the current impact of AI-assisted photosensitizer discovery. Many models are trained on small or chemically restricted datasets, and reported performance may be affected by data leakage, insufficient external validation, unbalanced classes, endpoint inconsistency, or a lack of applicability-domain analysis. Photodynamic outcomes are also highly context-dependent, influenced by solvent, pH, aggregation state, irradiation wavelength, light dose, oxygen availability, formulation, incubation time, biological model, and endpoint measurement. Future studies should therefore report model architecture, training data, validation strategy, performance metrics, uncertainty estimates, and applicability domain in a transparent manner. For natural photosensitizers, curated datasets integrating molecular structure, photophysical properties, ROS generation, singlet oxygen yield, phototoxicity, dark toxicity, formulation parameters, and biological endpoints will be essential for transforming AI from a screening tool into a reliable translational development strategy [23,30,31,32,33].

### 5.2. AI-Assisted Pharmaceutical Development and Nanotechnology

The therapeutic performance of PDT depends not only on the intrinsic photochemical properties of the photosensitizer but also on its pharmaceutical behavior, including solubility, chemical and photochemical stability, aggregation state, biodistribution, cellular uptake, tissue accumulation, retention, release profile, and compatibility with the intended route of administration. These aspects are particularly important for natural photosensitizers, many of which exhibit poor aqueous solubility, limited stability, variable purity, aggregation tendency, low bioavailability, and suboptimal pharmacokinetic or tissue-distribution profiles. Therefore, formulation design should not be viewed as a late-stage technical adjustment, but as a central determinant of whether natural photoactive compounds can become clinically relevant PDT platforms [16,17,30,48,49,52].

Nanotechnology-based delivery systems have been widely explored to overcome these limitations, but their translational value depends on more than improved in vitro phototoxicity. Hypericin-loaded glyconanoparticles, targeted hypericin nanoparticles, and polydopamine-coated cerium oxide nanorods illustrate how nanocarriers can improve dispersion, targeting, uptake, imaging potential, and ROS-mediated therapeutic performance [49,51,52]. Similarly, hypocrellin-HSA assemblies and curcumin-containing nanogels demonstrate how carrier design can modulate photophysical behavior, biological localization, and topical or phototheranostic performance [16,48,50]. However, these systems also highlight a recurring limitation: formulation studies frequently report particle characterization and biological efficacy but provide limited mechanistic modeling of how formulation variables influence optical behavior, release, stability, tissue interaction, and therapeutic outcome.

For AI-driven nanopharmaceutical development, the most relevant formulation variables include carrier type, polymer or lipid composition, surfactant concentration, particle size, polydispersity index, zeta potential, encapsulation efficiency, drug-loading capacity, photosensitizer aggregation state, release kinetics, rheological behavior, bioadhesion, tissue penetration or retention, photostability, sterilization compatibility, and storage stability. These variables are interdependent and difficult to optimize using conventional one-factor-at-a-time experimentation. ML models, artificial neural networks, random forests, support vector machines, Gaussian process models, and Bayesian optimization could be used to identify nonlinear relationships among formulation composition, physicochemical properties, irradiation parameters, and biological outcomes. Such approaches could complement design-of-experiments and quality-by-design strategies, reducing empirical screening and improving formulation robustness [18,21,30,31].

The practical impact of AI in this area would be particularly strong for natural photosensitizers because their pharmaceutical limitations are multidimensional. For example, curcumin formulations require optimization of stability, topical retention, degradation control, and light exposure; hypericin systems require aggregation control, solubilization, and selective delivery; and hypocrellin platforms require carrier-mediated biodistribution, imaging compatibility, and performance under complex tumor microenvironments. AI-assisted formulation models could prioritize carrier systems according to the specific limitation of each photosensitizer rather than applying generic nanocarrier strategies. This would support a more rational connection between molecular properties, delivery-system design, and intended clinical application [16,17,48,49,50,52].

The convergence between AI and nanotechnology is also important for multifunctional PDT platforms, in which photosensitizer delivery, imaging, targeting, controlled release, oxygen modulation, photothermal effects, immune activation, and therapeutic monitoring may be integrated into a single system. Predictive models could assist in selecting carrier materials, optimizing process parameters, estimating formulation stability, anticipating protein corona formation or biological interactions, and identifying the most relevant critical quality attributes for PDT performance. Nevertheless, current evidence remains mostly conceptual or experimentally driven, and few studies provide validated AI models that directly guide nanoformulation design for natural photosensitizers. This gap limits the ability to translate promising nanosystems into reproducible pharmaceutical products [30,31,49,50,52].

A major barrier to AI-assisted formulation design is the scarcity of standardized datasets linking nanocarrier composition, preparation method, physicochemical characterization, photosensitizer loading, aggregation state, release profile, irradiation conditions, biological model, and therapeutic endpoints. For natural photosensitizers, this limitation is amplified by variability in extraction source, chemical purity, batch composition, degradation profile, and formulation method. Future studies should therefore report formulation and irradiation parameters in a standardized manner and create curated databases that include both successful and unsuccessful formulations. Such datasets would allow AI models to identify critical formulation attributes, predict failure modes, and connect nanotechnological parameters with PDT efficacy, safety, manufacturability, and translational feasibility [17,30,31,48].

### 5.3. AI in Precision Photodynamic Therapy: Radiomics, Imaging, and Outcome Prediction

Beyond photosensitizer discovery and formulation development, AI is increasingly relevant for precision PDT because therapeutic outcomes depend on multiple variables that interact across molecular, pharmaceutical, tissue, and patient levels. These variables include photosensitizer accumulation, formulation-dependent biodistribution, tissue oxygenation, optical penetration, light dose, tumor architecture, vascular response, inflammatory or immune effects, and biological heterogeneity. Conventional statistical approaches may be insufficient to integrate these multidimensional data streams, whereas AI-based models can potentially identify nonlinear relationships across imaging, clinical, optical, formulation, and treatment-related datasets. However, the clinical value of such models depends on robust validation, interpretability, and evidence that predictions can meaningfully improve treatment planning or patient outcomes [19,20,31,36,38].

Radiomics represents one of the most relevant AI-related strategies for precision PDT because it converts medical images into quantitative features that may reflect tissue structure, spatial heterogeneity, vascular changes, and treatment-induced biological effects. In the PDT context, radiomics can support non-invasive assessment of tumor response, photodynamic priming, patient stratification, and therapy monitoring. Vincent et al. [38] evaluated computed tomography (CT) radiomic features associated with photodynamic priming in pancreatic cancer, providing an important example of how image-derived biomarkers can capture PDT-induced tissue modifications. A key strength of this approach is its potential to extract information from standard clinical imaging; however, radiomic models remain sensitive to image acquisition protocols, segmentation methods, feature extraction pipelines, cohort size, and validation strategy. These factors must be standardized before radiomics can be translated into reliable PDT decision support [19,38].

AI-guided imaging can also contribute to real-time procedural assistance and lesion detection. Rivera-Piza et al. [35] reported a real-time AI-guided PDT laparoscopy strategy for peritoneal cancer detection, highlighting the potential of computer-assisted image interpretation to improve lesion visualization and intraoperative decision-making. This application is particularly relevant when small lesions, diffuse disease, variable tissue fluorescence, or heterogeneous photosensitizer distribution compromise visual detection. Nevertheless, AI-guided procedural tools require rigorous evaluation under clinically realistic conditions, including variable illumination, tissue motion, autofluorescence, camera systems, surgeon interaction, and false-positive or false-negative detection rates. Without such validation, improved image detection may not necessarily translate into improved therapeutic precision or clinical benefit [31,35].

Outcome prediction is another major component of precision PDT. Zhu et al. [36] developed a multimodal DL model to predict PDT outcomes in patients with port-wine stains treated with hematoporphyrin monomethyl ether (HMME)-mediated PDT. By integrating clinical and imaging-related information, this study illustrates how AI may support individualized response prediction and treatment planning. Similar predictive frameworks have been explored in contextual studies involving chronic central serous chorioretinopathy and oral leukoplakia, where DL models were used to estimate therapeutic response, short-term efficacy, or recurrence risk after PDT. The main limitation of these models is that many are developed using restricted datasets, retrospective cohorts, or single-center experiences, which limits generalizability. Future studies should include external validation, calibration analysis, explainability, clinically meaningful endpoints, and prospective testing before outcome prediction models are incorporated into routine PDT workflows [27,28,29,36].

AI may also support PDT dosimetry and treatment monitoring, which remain major barriers to clinical standardization. Effective PDT requires coordinated control of photosensitizer concentration, light dose, fluence rate, tissue optical properties, oxygen availability, and treatment geometry. ML-based models can potentially estimate optical parameters, predict light distribution, identify under-treated regions, and support adaptive treatment planning. For example, simulation-based ML approaches have been used for real-time optical property recovery in interstitial PDT, suggesting that AI may contribute to more accurate light delivery and treatment control. However, simulation-trained models must be validated against experimental and clinical datasets, because biological tissues present heterogeneity, motion, perfusion changes, and oxygen dynamics that may not be fully captured by simplified computational environments [20,26,31].

For natural photosensitizer-based nanopharmaceuticals, precision PDT models should also incorporate formulation-specific variables. Nanocarriers can alter photosensitizer distribution, release, cellular uptake, fluorescence intensity, optical response, clearance, and tissue retention. Consequently, imaging and outcome prediction models developed for free photosensitizers or conventional agents may not be directly transferable to nanostructured natural photosensitizer systems. Future AI models should integrate formulation descriptors, photophysical properties, irradiation conditions, imaging features, biological response, and clinical endpoints into unified datasets. This would allow precision PDT to account not only for patient heterogeneity but also for the pharmaceutical behavior of the administered photosensitizer platform [30,31,48,49,50].

Taken together, these studies indicate that AI can extend PDT from a protocol-driven intervention toward a more personalized and data-guided therapeutic strategy. At present, however, the evidence is stronger for technical feasibility than for validated clinical implementation. Major limitations include small datasets, heterogeneous imaging and irradiation protocols, limited external validation, insufficient interpretability, lack of prospective studies, and incomplete integration of molecular, formulation, optical, and clinical variables. Future studies should prioritize multicenter datasets, standardized imaging and irradiation parameters, transparent model reporting, explainable AI methods, prospective validation, and clinically actionable endpoints. These advances will be essential for integrating radiomics, AI-guided imaging, dosimetry modeling, and outcome prediction into reliable precision PDT workflows [31,35,36,38].

### 5.4. Practical Requirements for Translating AI Models into PDT Nanopharmaceutical Development

For AI to have a practical impact in PDT nanopharmaceutical development, computational models must be connected to experimentally measurable and pharmaceutically meaningful endpoints. In molecular discovery, this requires standardized reporting of absorption spectra, fluorescence behavior, photostability, ROS generation, singlet oxygen quantum yield, dark toxicity, phototoxicity, and structure–property relationships. In formulation development, AI models should incorporate particle size, polydispersity, surface charge, encapsulation efficiency, drug loading, release kinetics, aggregation state, rheological properties, stability, and biological performance. In clinical translation, predictive systems should integrate imaging features, light-delivery parameters, oxygen status, photosensitizer distribution, treatment response, and patient-specific variables. Without this alignment between computational inputs and experimental or clinical outputs, AI models may remain technically interesting but difficult to implement in reproducible PDT workflows [23,30,31,32,33].

Another practical requirement is model validation beyond internal performance metrics. Many AI studies report accuracy, regression performance, or classification scores, but translational relevance depends on external validation, applicability-domain definition, uncertainty estimation, explainability, and prospective testing. For natural photosensitizer-based nanopharmaceuticals, validation should also consider formulation-dependent behavior, because the same photosensitizer may exhibit different photophysical and biological responses depending on carrier composition, aggregation state, release profile, and tissue localization. Therefore, future AI-assisted PDT studies should report not only predictive performance but also whether model-guided decisions improve candidate selection, formulation robustness, experimental efficiency, or therapeutic outcomes compared with conventional empirical workflows [30,31,35,36,38].

A final requirement is the integration of AI with pharmaceutical development principles. Design-of-experiments, quality-by-design, critical quality attributes, manufacturability, batch reproducibility, storage stability, sterilization compatibility, and regulatory-oriented comparability should be incorporated into AI-driven workflows whenever the goal is translational nanopharmaceutical development. This is particularly important for natural photosensitizers, whose source variability, chemical instability, hydrophobicity, and formulation dependence may compromise reproducibility. Connecting AI models with these pharmaceutical criteria would allow computational tools to support not only discovery but also the development of scalable, stable, safe, and clinically relevant PDT products [16,30,48,49,52].

## 6. Challenges, Limitations, and Future Perspectives

Despite the growing interest in natural photosensitizers and AI-assisted PDT, the field still faces several scientific, pharmaceutical, computational, and translational challenges that limit progression from experimental promise to clinically useful nanopharmaceutical platforms. Natural photosensitizers offer structural diversity, biological multifunctionality, and potential biocompatibility, but many candidates remain constrained by poor aqueous solubility, chemical and photochemical instability, aggregation, low bioavailability, limited tissue selectivity, variable photodynamic efficiency, and incomplete formulation readiness. These barriers are particularly evident for curcumin, hypericin, and hypocrellin-based systems, whose therapeutic value depends strongly on carrier design, irradiation parameters, route of administration, biological context, and reproducibility of pharmaceutical performance [16,17,48,49,50].

A central limitation is the fragmentation and heterogeneity of experimental data. Studies differ substantially in photosensitizer source, chemical purity, formulation method, concentration, incubation time, irradiation wavelength, fluence, fluence rate, oxygen availability, biological model, and endpoint measurement. This variability makes direct comparison difficult and weakens the ability to identify robust structure–property, formulation–performance, and treatment–response relationships. For AI applications, this problem is even more critical because predictive models require curated, consistent, and sufficiently large datasets. Models trained on heterogeneous or poorly annotated data may show acceptable internal performance but fail when applied to new photosensitizers, formulations, biological models, or clinical settings [23,30,31,32].

A related issue is the level of data saturation required for AI-assisted PDT models to achieve sufficient reliability. At present, no universal numerical threshold can be defined, because the amount of data required depends on the specific task, endpoint, model complexity, chemical domain, formulation space, and intended translational use. For molecular property prediction, data saturation should be assessed according to the chemical diversity of photosensitizers, the balance between active, weakly active, and inactive compounds, and the stability of predictive performance across independent test sets. For nanoformulation optimization, saturation requires adequate coverage of carrier composition, particle size, surface charge, loading capacity, release behavior, stability, irradiation parameters, and biological outcomes. For radiomics, dosimetry, and outcome-prediction models, saturation also depends on cohort size, imaging variability, treatment protocols, and multicenter external validation. Therefore, rather than relying on a fixed sample number, AI-assisted PDT studies should demonstrate saturation through learning-curve analysis, performance plateaus after addition of new data, narrow confidence intervals, calibrated uncertainty, stable results across repeated validation procedures, and clearly defined applicability domains. Models should be considered sufficiently reliable only when additional data no longer substantially improve performance and when external validation confirms that predictions remain robust across new compounds, formulations, biological models, or clinical datasets [18,21,23,30,31,32,35,36,37,38].

Another important concern is the risk of AI-related data hallucination or unsupported model outputs in PDT applications, particularly when AI systems are used for treatment-response prediction or clinical decision support. In this context, hallucination should not be understood only as a problem of generative AI producing fabricated text, but more broadly as the generation of plausible yet unreliable predictions, associations, explanations, or therapeutic recommendations that are not adequately supported by the underlying data. This risk is especially relevant in PDT because treatment outcomes depend on multiple interacting variables, including photosensitizer type, formulation, dose, irradiation wavelength, fluence, fluence rate, oxygen availability, lesion characteristics, imaging parameters, and patient-specific biological factors. Models trained on small, biased, retrospective, or poorly annotated datasets may therefore produce overconfident predictions that appear clinically meaningful but fail when applied to new patients, formulations, or treatment protocols. To reduce this risk, AI-assisted PDT models should incorporate uncertainty estimation, calibration analysis, external and prospective validation, out-of-distribution detection, explainable model outputs, and human expert oversight. Treatment outcome prediction models should be used as decision-support tools rather than autonomous decision-makers until their reliability, generalizability, and clinical utility have been demonstrated across independent and clinically realistic datasets [27,28,29,30,31,35,36,38].

Another major challenge is the limited integration between natural photosensitizer research and AI-assisted molecular discovery. Although ML and QSAR/QSPR approaches have shown promise for predicting photodynamic activity, singlet oxygen generation, phototoxicity, and photosensitizing effects, most available models are based on restricted chemical datasets and are rarely optimized specifically for natural-product-derived scaffolds. This creates a risk of chemical-space bias, in which models perform well for well-represented synthetic classes but poorly for complex natural structures. Future predictive datasets should include curcumin, hypericin, hypocrellin, chlorins, flavonoids, anthraquinones, pheophorbides, furanocoumarins, alkaloids, and marine-derived pigments, together with negative or weakly active examples. The inclusion of inactive compounds is particularly important for avoiding overly optimistic models and for defining meaningful applicability domains [12,14,15,23,33,37].

Pharmaceutical development and nanocarrier optimization represent equally important bottlenecks. Nanotechnology-based systems have improved the solubility, stability, biodistribution, targeting, and photodynamic efficacy of several natural photosensitizers, but most studies remain preclinical and empirically optimized. Formulation performance is influenced by interacting variables such as carrier composition, particle size, polydispersity, surface charge, encapsulation efficiency, loading capacity, aggregation state, release kinetics, rheology, bioadhesion, tissue penetration, biological interactions, and irradiation conditions. AI-assisted formulation optimization could help identify critical quality attributes and reduce trial-and-error experimentation, but this will require standardized datasets linking formulation variables with photophysical behavior, release profiles, biological response, safety, and manufacturability [30,48,49,50,51,52].

A further limitation is the insufficient connection between AI modeling and pharmaceutical translation. For a nanopharmaceutical PDT system to progress toward clinical use, predictive performance alone is not enough. Candidate systems must also demonstrate reproducible preparation, batch-to-batch consistency, long-term stability, sterility or sterilization compatibility, scalable manufacturing, defined critical quality attributes, safety under dark and irradiated conditions, and compatibility with clinically realistic light-delivery protocols. These aspects are rarely integrated into AI-PDT studies. Future work should therefore combine AI with design-of-experiments, quality-by-design principles, standardized physicochemical characterization, and translational comparability studies to ensure that computational optimization generates pharmaceutically viable products rather than only experimentally interesting prototypes [16,30,31,48].

The clinical implementation of AI-assisted PDT also faces important barriers. Radiomics, DL, image-guided therapy, dosimetry modeling, and outcome prediction depend on robust validation, reproducibility, interpretability, and integration with clinical workflows. Many current studies are limited by small sample sizes, single-center datasets, retrospective designs, and limited external validation. Differences in imaging protocols, segmentation methods, light-delivery parameters, photosensitizer formulation, treatment schedules, and outcome definitions may reduce model generalizability. Future AI-based PDT studies should prioritize multicenter validation, prospective evaluation, transparent reporting, explainable models, clinically meaningful endpoints, and assessment of whether model-guided decisions improve patient outcomes compared with standard protocols [27,28,29,35,36,38].

An important future direction is the development of integrated AI-driven PDT platforms capable of connecting molecular prediction, natural-product chemical diversity, nanoformulation optimization, photophysical characterization, imaging analysis, dosimetry modeling, and clinical outcome prediction. Such platforms could support the selection of optimized photosensitizer candidates, guide carrier development, anticipate formulation failure, personalize irradiation protocols, and predict therapeutic response. For natural photosensitizers, this approach is particularly valuable because it can connect chemical diversity with formulation behavior and patient-specific treatment variables, thereby accelerating the transition from isolated experimental discovery to translational application [21,26,30,31,39].

**Figure 3:**
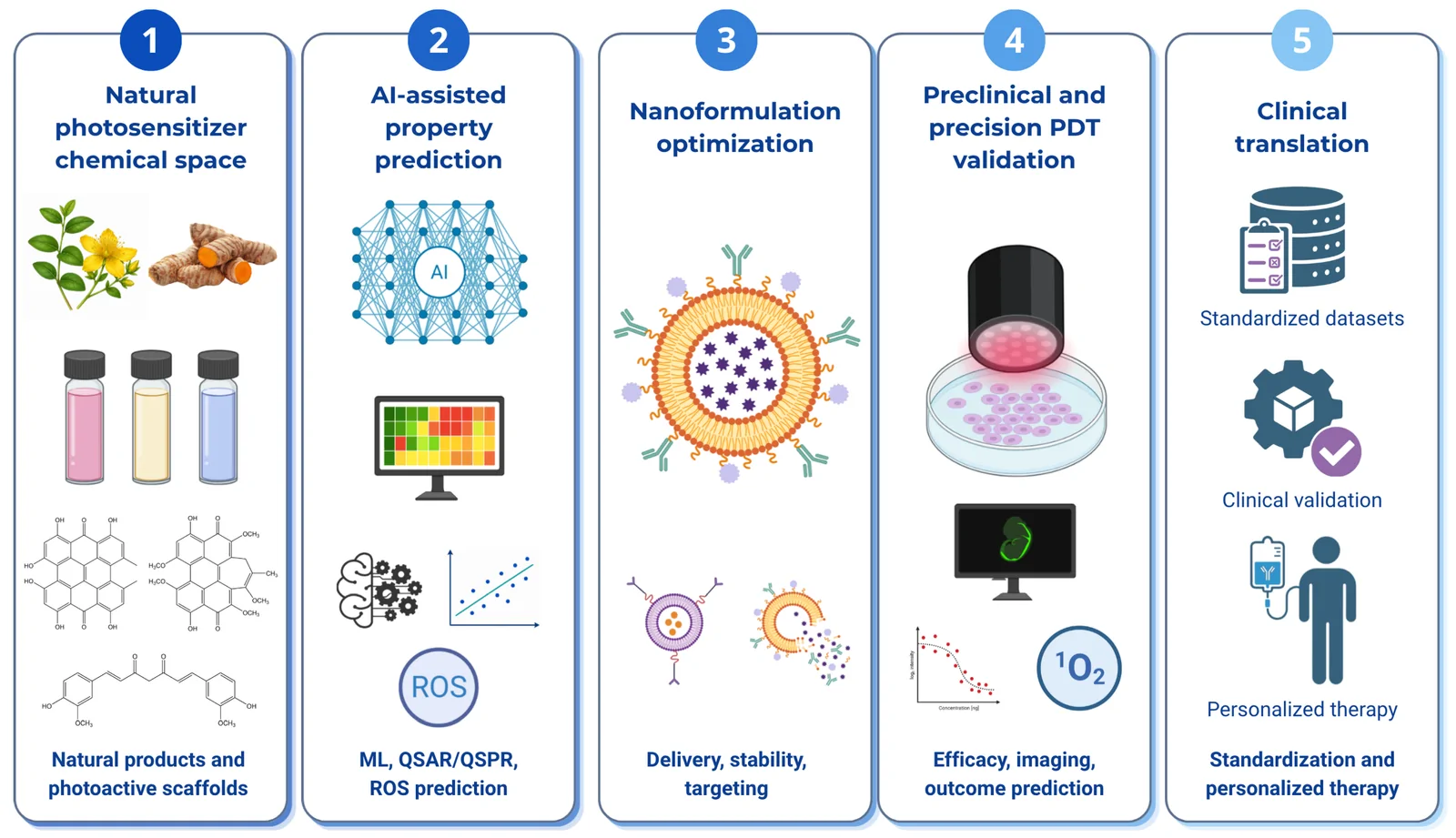
Integrated translational workflow for artificial intelligence-assisted development of natural photosensitizers in photodynamic therapy. The proposed framework connects five interdependent stages: natural photosensitizer chemical space, AI-assisted property prediction, nanoformulation optimization, preclinical and precision PDT validation, and clinical translation. Rather than representing a linear discovery pathway, the workflow emphasizes feedback between molecular prediction, formulation performance, biological validation, imaging, dosimetry, and clinical outcomes. This structure highlights the main translational bottlenecks identified in the review, including limited natural-product representation in predictive datasets, insufficient standardization of photophysical and formulation parameters, incomplete validation of AI models, and the need to connect nanopharmaceutical development with clinically realistic PDT protocols.

The integrated workflow proposed for the AI-assisted development of natural photosensitizers is illustrated in Figure 3. Rather than presenting future directions as isolated technical recommendations, Table 4 summarizes the main translational priorities that must be addressed to move AI-assisted natural photosensitizer-based PDT from descriptive research toward reproducible nanopharmaceutical development and clinical implementation.

These priorities include dataset standardization, expansion of natural-product representation in predictive models, integration of photochemical and photosafety prediction, AI-assisted nanoformulation optimization, precision PDT validation, and incorporation of manufacturing and regulatory considerations into AI-driven development workflows [23,30,31,32,33,36,38].

Overall, future progress will depend on closer integration between experimental PDT research, natural product chemistry, pharmaceutical technology, computational modeling, and clinical validation. The creation of open, standardized, and well-annotated datasets should be considered a priority because these resources would enable more robust AI models and more reproducible comparisons across studies. In parallel, natural photosensitizer research should move beyond isolated compound evaluation toward systematic workflows that combine molecular design, formulation engineering, mechanistic validation, manufacturing considerations, and precision treatment strategies. Such integration may substantially expand the therapeutic potential of natural photosensitizers and support the development of more effective, reproducible, personalized, and clinically translatable PDT nanopharmaceuticals [15,30,31,33,42].

This review also has limitations that should be acknowledged. Although a structured PRISMA 2020-inspired search strategy was adopted, the review was designed as a critical and translational synthesis rather than as a quantitative systematic review or meta-analysis. The search was restricted to PubMed and Scopus, and the final corpus was defined according to studies directly relevant to AI, natural photosensitizers, nanopharmaceutical development, and PDT. As a result, some studies related to adjacent fields, such as general AI-assisted drug discovery, broader nanomedicine optimization, photobiomodulation, conventional photosensitizer chemistry, or non-PDT phototoxicity models, may not have been included in the quantitative corpus. To mitigate this limitation, complementary seminal and recent references were incorporated for contextual interpretation, particularly when they contributed to mechanistic understanding, computational methodology, pharmaceutical development, dosimetry, or clinical translation. Nevertheless, the conclusions of this review should be interpreted as a critical synthesis of an emerging interdisciplinary field rather than as an exhaustive quantitative mapping of all AI or nanotechnology applications in PDT [24,25,30,31,34,39].

## 7. Conclusions

This critical review highlights the growing but still incomplete convergence between natural photosensitizers, AI-assisted methodologies, nanopharmaceutical development, and PDT. Natural compounds such as curcumin, hypericin, hypocrellin, chlorin-related systems, and other photoactive metabolites remain attractive because of their structural diversity, biological activity, and potential therapeutic applicability. However, their effective translation into PDT platforms is limited not only by photochemical constraints but also by pharmaceutical barriers, including poor solubility, instability, aggregation, low bioavailability, insufficient tissue selectivity, formulation complexity, limited reproducibility, and variable performance under different irradiation and biological conditions [12,13,14,15,16,40,41].

The evidence analyzed in this review indicates that AI can support multiple stages of the PDT workflow, including photosensitizer discovery, molecular property prediction, phototoxicity assessment, nanoformulation development, image-guided therapy, radiomics, dosimetry support, and treatment-response prediction. Nevertheless, these applications are not equally mature. Molecular property prediction, phototoxicity screening, and imaging-based outcome modeling currently provide the clearest examples of AI implementation, whereas AI-guided nanoformulation optimization and clinically validated decision-support systems remain comparatively underdeveloped. Therefore, the main contribution of AI at present is not the replacement of experimental development, but the prioritization, organization, and interpretation of complex datasets that can guide more rational experimental and translational workflows [23,31,32,33,35,36,38].

A central conclusion of this review is that the major limitation of the field is not the absence of promising photosensitizers or computational tools, but the weak integration among molecular prediction, formulation science, biological validation, and clinical translation. Most AI-based PDT studies are still based on limited datasets, heterogeneous experimental conditions, and insufficient external validation. At the same time, many natural photosensitizer studies remain experimentally driven and are not connected to predictive modeling pipelines. This disconnect prevents the construction of robust models capable of linking chemical structure, photophysical behavior, formulation characteristics, irradiation parameters, biological activity, imaging features, and clinical outcomes [23,30,31,42].

Future progress will require standardized, interdisciplinary, and translationally oriented research strategies. Curated datasets integrating molecular descriptors, singlet oxygen generation, ROS production, photostability, aggregation behavior, light parameters, formulation properties, release profiles, biological endpoints, imaging features, and clinical outcomes will be essential for developing reliable AI-assisted PDT models. For natural photosensitizers, such resources are particularly important because their chemical diversity remains underrepresented in predictive modeling and their clinical value depends on coordinated advances in molecular design, nanopharmaceutical development, quality-by-design strategies, precision therapy, and prospective validation [15,24,25,33,39].

Overall, AI-assisted approaches should be viewed as complementary tools that can increase efficiency, reproducibility, and translational relevance in PDT research. By integrating natural product chemistry, nanotechnology, computational modeling, formulation optimization, imaging analysis, dosimetry, and clinical validation, future studies may accelerate the development of more effective, selective, reproducible, and personalized PDT strategies. The most promising direction is the creation of AI-driven nanopharmaceutical workflows in which natural photosensitizer discovery, delivery-system optimization, treatment planning, and outcome prediction are developed as connected components of a single translational pipeline. Such integration may expand the therapeutic potential of natural photosensitizers and contribute to the broader advancement of PDT as a precision-oriented treatment modality [26,27,28,29,31,35,36].

## Figures and Tables

**Table 1:** Characteristics of studies included in the qualitative synthesis (*n* = 27).

Ref.	Year	AI Approach	PS/System	Main Contribution
**Review Articles**				
[12]	2018	–	Natural products	Reviewed natural products and derivatives as photosensitizers for PDT.
[40]	2019	–	Natural products	Discussed the role of natural compounds in cancer PDT.
[41]	2021	–	Natural PS	Reviewed natural photosensitizers for antimicrobial PDT.
[16]	2021	Indirect	Curcumin	Summarized curcumin-based innovations and nanotechnologies for PDT.
[13]	2022	–	Natural PS	Reviewed medicinal properties and PDT applications of natural photosensitizers.
[14]	2023	–	Natural products	Summarized potential natural photosensitizers in cancer PDT.
[15]	2024	–	Natural products	Reviewed natural-product-based photosensitizers and PDT applications.
[42]	2025	AI	Antimicrobial PDT	Discussed AI applications for improving antimicrobial PDT effectiveness.
[17]	2025	Indirect	Hypericin	Reviewed nanosized hypericin systems for ROS-mediated therapies.
[30]	2025	ML	Nano-enhanced PDT	Discussed machine learning and nano-enhanced PDT.
[31]	2026	AI	PDT	Reviewed current and future applications of AI in PDT.
**Original Studies**				
[33]	2025	ML	Cyanine PS	ML-assisted design of high-performance cyanine photosensitizers.
[35]	2025	AI-guided imaging	PDT laparoscopy	Real-time AI-guided PDT laparoscopy for peritoneal cancer detection.
[36]	2025	DL	HMME-PDT	Predicted port-wine stain PDT outcomes using multimodal deep learning.
[37]	2025	AI	Drug photosensitization	Predicted photosensitizing effects of drugs using AI.
[46]	2023	–	Chlorin e6-curcumin	Developed chlorin e6-curcumin derivatives with anticancer PDT activity.
[32]	2024	ML	Phototoxic compounds	Developed MLtox, an online platform for phototoxicity prediction.
[23]	2021	QSAR/QSPR	BODIPY PS	Modeled photodynamic activity and singlet oxygen generation.
[38]	2021	Radiomics	Pancreatic cancer PDT	Evaluated CT radiomic features of photodynamic priming.
[47]	2023	–	Hypericin	Evaluated hypericin photoactivity for antifungal applications.
[48]	2025	Nanoformulation	Curcumin nanogel	Developed curcumin and essential-oil nanogel for daylight PDT.
[53]	2024	–	Natural PS	Reduced multispecies biofilms using natural photosensitizer-mediated antimicrobial PDT.
[49]	2023	Nanotechnology	Hypericin nanoparticles	Developed targeted hypericin nanoparticles for melanoma theranostics.
[50]	2019	Nanotechnology	Hypocrellin-HSA	Developed NIR-responsive hypocrellin assembly for hypoxic tumors.
[54]	2020	Combination therapy	Catechin + phthalocyanine	Enhanced phthalocyanine-mediated PDT using catechin co-treatment.
[51]	2018	Nanotechnology	Hypericin glyconanoparticles	Fabricated targeted hypericin-loaded glyconanoparticles.
[52]	2019	Nanotechnology	Hypericin delivery	Developed polydopamine-coated cerium oxide nanorods for hypericin delivery.

**Table 2:** Comparative translational profile of major natural photosensitizer classes discussed in this review.

Photosensitizer Class	Main Advantages	Key Pharmaceutical Limitations	AI/Nanopharmaceutical Priority
Curcumin-based systems	Low dark toxicity, broad biological activity, topical applicability, and compatibility with combination strategies.	Poor aqueous solubility, rapid degradation, low bioavailability, and limited tissue penetration due to blue-light activation.	Optimize curcumin, natural curcuminoids such as BDMC, derivatives, and topical nanoformulations using models that integrate hydrophilicity, chemical stability, degradation behavior, release, retention, phototoxicity, and formulation performance [16,43,44,45,46,48].
Hypericin-based systems	High photodynamic potency, strong fluorescence, phototheranostic potential, and relevance for imaging-guided PDT.	Hydrophobicity, aggregation in biological media, limited bioavailability, and need for selective delivery.	Model aggregation behavior, carrier compatibility, fluorescence response, ROS generation, targeting, and image-guided therapeutic outcomes [17,47,49,51,52].
Hypocrellin-based systems	Favorable visible absorption, efficient ROS generation, photostability, and potential for phototheranostic platforms.	Poor water solubility, aggregation, limited tumor accumulation, and comparatively lower translational maturity.	Develop predictive workflows connecting molecular properties, protein or nanocarrier binding, biodistribution, imaging behavior, and NIR-responsive performance [15,50].
Chlorophyll derivatives and pheophorbides	Relevant absorption properties, natural abundance, and structural relationship with clinically established porphyrinoid photosensitizer families.	Variable stability, hydrophobicity, formulation dependence, and limited standardized comparison across biological models.	Expand AI-assisted screening beyond dominant scaffolds and connect photophysical prediction with delivery-system design and standardized PDT endpoints [12,14,15].
Other emerging natural scaffolds	Broad chemical diversity, potential multifunctional biological activity, and access to underexplored photoactive structures.	Limited photophysical characterization, source variability, low availability, and scarce formulation or translational data.	Integrate biodiversity databases, molecular descriptors, photochemical screening, nanoformulation compatibility, and negative examples into curated AI-ready datasets [12,13,14,15].

Abbreviations: AI, artificial intelligence; BDMC, bisdemethoxycurcumin; NIR, near-infrared; PDT, photodynamic therapy; ROS, reactive oxygen species.

**Table 3:** Analytical summary of artificial intelligence applications, current bottlenecks, and translational priorities in photodynamic therapy.

Analytical Dimension	Critical Synthesis
**Photosensitizer discovery and molecular design**	
Approaches	ML models, molecular descriptors, molecular fingerprints, graph-based learning, and generative approaches.
Current bottleneck	Small and chemically biased datasets, with limited representation of natural-product-derived scaffolds.
Translational priority	Curate datasets including synthetic and natural photosensitizers, active and inactive compounds, and standardized photophysical endpoints [31,33].
**Photochemical property prediction**	
Approaches	QSAR, QSPR, DFT–ML workflows, and regression models.
Current bottleneck	Predictions depend strongly on solvent, aggregation state, oxygen availability, irradiation wavelength, light dose, and chemical class.
Translational priority	Define applicability domains and validate predictions using standardized singlet oxygen, ROS, and photostability assays [23,24,25].
**Photosafety assessment**	
Approaches	Phototoxicity classifiers and drug photosensitization models.
Current bottleneck	Phototoxicity prediction does not necessarily indicate therapeutic usefulness, because PDT suitability also depends on selectivity, dark toxicity, delivery, and formulation compatibility.
Translational priority	Combine photosafety prediction with dark toxicity, biological selectivity, formulation compatibility, and delivery-performance criteria [32,37].
**Nanoformulation and pharmaceutical optimization**	
Approaches	ML-assisted formulation analysis, design-of-experiments-integrated models, and Bayesian optimization.
Current bottleneck	Few datasets connect nanocarrier composition, particle size, surface charge, loading, release, stability, safety, efficacy, and manufacturability with PDT outcomes.
Translational priority	Develop formulation databases linking critical quality attributes, photosensitizer behavior, irradiation parameters, biological response, safety, scalability, and translational feasibility [30,48,49,52].
**Image-guided PDT and radiomics**	
Approaches	Computer vision, fluorescence image analysis, AI-guided imaging, and CT radiomics.
Current bottleneck	Model performance is sensitive to image acquisition protocol, segmentation method, illumination, tissue motion, autofluorescence, and cohort size.
Translational priority	Standardize imaging workflows and validate algorithms under multicenter and clinically realistic conditions [19,35,38].
**Outcome prediction and dosimetry support**	
Approaches	DL, multimodal learning, simulation-based ML, predictive dosimetry, and clinical decision-support models.
Current bottleneck	Retrospective datasets, single-center cohorts, limited explainability, and scarce prospective validation.
Translational priority	Develop explainable and prospectively validated models incorporating optical, clinical, imaging, treatment-related, and formulation-specific variables [26,27,28,29,36].

Abbreviations: AI, artificial intelligence; CT, computed tomography; DFT, density functional theory; DL, deep learning; ML, machine learning; PDT, photodynamic therapy; QSAR, quantitative structure–activity relationship; QSPR, quantitative structure–property relationship; ROS, reactive oxygen species.

**Table 4:** Critical translational priorities for AI-assisted natural photosensitizer-based photodynamic therapy.

Priority Area	Critical Interpretation and Future Direction
Dataset standardization	The major obstacle for robust AI models is the heterogeneity of PDT datasets. Studies differ in photosensitizer source, purity, concentration, formulation, irradiation wavelength, fluence, fluence rate, oxygen availability, biological model, and endpoint definition. Future datasets should report these variables systematically and include standardized photophysical, formulation, biological, and irradiation parameters to enable reproducible model training and external validation [23,30,31,32].
Natural-product chemical space	Current AI-assisted PDT research remains biased toward a limited number of photosensitizer classes, whereas natural-product-derived scaffolds are still underrepresented. This restricts model generalizability and may prevent the identification of promising underexplored compounds. Future workflows should include curcumin, hypericin, hypocrellin, chlorins, pheophorbides, flavonoids, anthraquinones, furanocoumarins, alkaloids, marine pigments, and inactive or weakly active examples to reduce chemical-space bias [12,14,15,33,37].
Photochemical and photosafety prediction	Prediction of ROS generation, singlet oxygen quantum yield, photostability, aggregation behavior, dark toxicity, and phototoxicity remains central to AI-assisted photosensitizer development. However, these endpoints are highly dependent on solvent, formulation, irradiation conditions, and biological context. Future models should combine QSAR/QSPR, DFT–ML, experimental validation, and applicability-domain analysis to support more reliable candidate prioritization [23,24,25,32,37].
Nanoformulation optimization	Natural photosensitizers often require nanocarriers to overcome poor solubility, instability, aggregation, limited bioavailability, and insufficient tissue selectivity. AI can support formulation optimization only if datasets connect carrier composition, particle size, surface charge, loading, release, stability, rheology, bioadhesion, efficacy, safety, and manufacturability. Future studies should integrate AI with design-of-experiments and quality-by-design strategies to move beyond empirical formulation screening [30,48,49,50,51,52].
Precision PDT and clinical validation	Radiomics, image-guided PDT, dosimetry modeling, and outcome prediction may support patient stratification and personalized treatment planning. Nevertheless, most models remain limited by small cohorts, retrospective designs, single-center datasets, and insufficient explainability. Future studies should prioritize multicenter validation, prospective evaluation, standardized imaging and irradiation protocols, clinically meaningful endpoints, and demonstration that AI-guided decisions improve therapeutic outcomes [27,28,29,35,36,38].
Manufacturing and regulatory translation	A recurring translational gap is the limited connection between AI-based optimization and pharmaceutical product development. For clinical translation, natural photosensitizer nanopharmaceuticals must demonstrate reproducible preparation, batch-to-batch consistency, long-term stability, sterilization compatibility, scalable manufacturing, defined critical quality attributes, and safety under dark and irradiated conditions. Future AI-driven workflows should incorporate manufacturability, quality control, and regulatory-oriented comparability as development endpoints [16,30,31,48].

*Abbreviations:* AI, artificial intelligence; DFT, density functional theory; ML, machine learning; PDT, photodynamic therapy; QSAR, quantitative structure–activity relationship; QSPR, quantitative structure–property relationship; ROS, reactive oxygen species.

## Data Availability

No new data were created or analyzed in this study. Data sharing is not applicable to this article.

## Abbreviations

The following abbreviations are used in this manuscript:

AI	Artificial intelligence
aPDT	Antimicrobial photodynamic therapy
BDMC	Bisdemethoxycurcumin
BODIPY	Boron-dipyrromethene
CT	Computed tomography
DFT	Density functional theory
DL	Deep learning
DMC	Demethoxycurcumin
DoE	Design of experiments
HMME	Hematoporphyrin monomethyl ether
HSA	Human serum albumin
ML	Machine learning
NIR	Near-infrared
PDT	Photodynamic therapy
PRISMA	Preferred Reporting Items for Systematic Reviews and Meta-Analyses
PS	Photosensitizer
QbD	Quality by design
QSAR	Quantitative structure–activity relationship
QSPR	Quantitative structure–property relationship
ROS	Reactive oxygen species
